# Supplementary material for: Resemblance of nutrient intakes in three generations of parent-offspring pairs: Tehran lipid and Glucose Study
Source: PLoS One. 2022 Apr 15;17(4):e0266941. doi: 10.1371/journal.pone.0266941 (PMC9012390; doi:10.1371/journal.pone.0266941)
Supplement: S1 Table — (DOCX) [file pone.0266941.s001.docx]

**Supplementary Table 1. Linear regression model for predicting offspring dietary intakes by differences in living arrangements**

|  | **Offspring**  **(living with their parents)** | | **Offspring**  **(living independent of their parents)** | |
| --- | --- | --- | --- | --- |
|  | **β** | **P** | **β** | **P** |
| **n** (paired) | 2358 | | 1317 | |
| Total energy (Kcal/day) | 0.14 | <0.001 | 0.06 | 0.02 |
| Carbohydrate ^a^ | 0.12 | <0.001 | 0.21 | <0.001 |
| Protein ^a^ | 0.22 | <0.001 | 0.08 | <0.001 |
| Total fat ^a^ | 0.19 | <0.001 | 0.14 | <0.001 |
| SFA ^a^ | 0.19 | <0.001 | 0.07 | 0.005 |
| Trans-fatty acids ^a^ | 0.21 | <0.001 | 0.07 | 0.03 |
| MUFA ^a^ | 0.20 | <0.001 | 0.06 | 0.001 |
| PUFA ^a^ | 0.24 | <0.001 | 0.08 | <0.001 |
| Fiber ^b^ | 0.22 | <0.001 | 0.08 | 0.04 |
| Cholesterol (mg/day) | 0.07 | <0.001 | 0.06 | 0.02 |
| Vitamin C ^c^ | 0.18 | <0.001 | 0.05 | 0.07 |
| Calcium ^c^ | 0.20 | <0.001 | 0.01 | 0.58 |
| Iron ^c^ | 0.21 | <0.001 | -0.02 | 0.29 |
| Zinc ^c^ | 0.24 | <0.001 | 0.03 | 0.10 |
| Sodium (mg/day) | 0.15 | <0.001 | 0.02 | 0.39 |
| Magnesium ^c^ | 0.22 | <0.001 | 0.06 | 0.02 |

Linear regression analysis (main exposure: parent’s dietary intake, adjusted for age, physical activity and body mass index)

^a^ (% of energy intake), ^b^ gr/1000 Kcal/day, ^c^ mg/1000 Kcal/day, P<0.01 is considered to be significant based on false discovery rate.

β: Regression coefficient, SFA: Saturated fatty acid; MUFA: Mono-unsaturated fatty acid; PUFA: Poly unsaturated fatty acid
