# Supplementary material for: Resemblance of nutrient intakes in three generations of parent-offspring pairs: Tehran lipid and Glucose Study
Source: PLoS One. 2022 Apr 15;17(4):e0266941. doi: 10.1371/journal.pone.0266941 (PMC9012390; doi:10.1371/journal.pone.0266941)
Supplement: S2 Table — (DOCX) [file pone.0266941.s002.docx]

**Supplementary Table 2. Linear regression model for predicting grandson/daughter dietary intakes**

|  | **Grandson/daughter** | |
| --- | --- | --- |
|  | **β** | **P** |
| **n** (paired) |  | |
| Total energy (Kcal/day) | 0.06 | 0.44 |
| Carbohydrate ^a^ | 0.05 | 0.27 |
| Protein ^a^ | 0.09 | 0.002 |
| Total fat ^a^ | 0.07 | 0.17 |
| SFA ^a^ | 0.05 | 0.31 |
| Trans-fatty acids ^a^ | 0.04 | 0.48 |
| MUFA ^a^ | 0.05 | 0.07 |
| PUFA ^a^ | 0.12 | 0.008 |
| Fiber ^b^ | 0.08 | 0.05 |
| Cholesterol (mg/day) | 0.10 | 0.23 |
| Vitamin C ^c^ | 0.07 | 0.09 |
| Calcium ^c^ | 0.09 | 0.30 |
| Iron ^c^ | 0.02 | 0.53 |
| Zinc ^c^ | 0.04 | 0.02 |
| Sodium (mg/day) | 0.03 | 0.46 |
| Magnesium ^c^ | 0.07 | 0.10 |

Linear regression analysis (main exposure: grandparents’ dietary intake, adjusted for age, physical activity and body mass index)

^a^ (% of energy intake), ^b^ gr/1000 Kcal/day, ^c^ mg/1000 Kcal/day, P<0.01 is considered to be significant based on false discovery rate.

SFA: Saturated fatty acid; MUFA: Mono-unsaturated fatty acid; PUFA: Poly unsaturated fatty acid
